# DNASE1L3 arrests tumor angiogenesis by impairing the senescence-associated secretory phenotype in response to stress

**DOI:** 10.18632/aging.202740

**Published:** 2021-03-19

**Authors:** Deliang Guo, Dong Ma, Pengpeng Liu, Jianwei Lan, Zhisu Liu, Quanyan Liu

**Affiliations:** 1 Department of Hepatobiliary Surgery, Zhongnan Hospital of Wuhan University, Wuhan 430071, P.R. China; 2 Department of Hepatobiliary Surgery, Tianjin Medical University General Hospital, Tianjin 300052, P.R. China

**Keywords:** senescence, angiogenesis, hepatocellular carcinoma, DNASE1L3

## Abstract

Hepatocellular carcinoma (HCC) is one of the most challenging and aggressive cancers with limited treatment options because of tumor heterogeneity. Tumor angiogenesis is a hallmark of HCC and is necessary for tumor growth and progression. DNA damage stress and its associated deoxyribonuclease1-like 3 (DNASE1L3) are involved in HCC progression. Here, we explored the influence mechanism of DNASE1L3 on tumor angiogenesis under DNA damage stress *in vitro* and *in vivo*. DNASE1L3 was found downregulated and negatively correlated with poor prognosis of resectable and unresectable HCC patients. The tissue microarray of HCC revealed the negative association between DNASE1L3 and cancer vasculature invasion. Mechanistically, DNASE1L3 was found to relieve cytoplasmic DNA accumulation under DNA damage stress in HCC cell lines, in turn cell senescence and senescence-associated secretory phenotype were arrested via the p53 and NF-κB signal pathway, and hence, tumor angiogenesis was impaired. Furthermore, we found that DNASE1L3 excised these functions by translocating to the nucleus and interacting with H2BE under DNA damage stress using co-immunoprecipitation and fluorescence resonance energy transfer assay. In conclusion, DNASE1L3 inhibits tumor angiogenesis via impairing the senescence-associated secretory phenotype in response to DNA damage stress.

## INTRODUCTION

Hepatocellular carcinoma (HCC) is one of the most common digestive malignancies worldwide. Although treatments such as radical surgery, chemotherapy, radiotherapy and target therapy are of value in the management of these tumors, the prognosis of patients diagnosed with HCC remains grave. Thus, it is still a major risk factor threatening human health [1–5]. Multiple factors have been confirmed in the etiology of HCC, including hepatitis B or C virus (HBV or HCV) infections, obesity, alcohol consumption and dietary pollution [6]. These factors are also associated with chronic liver damage and inflammation, which can promote DNA damage and chromosome aberrations, as well as trigger a series of signaling pathways. One major downstream signaling route is via the DNA damage response (DDR) pathway, which is involved in DNA repair regulation and cell cycle arrest, eventually leading to cell death or senescence [7]. Meanwhile, DDR aberrations can destroy genomic integrity, trigger liver cancer pathogenesis, and promote the development of advanced HCC [8]. Therefore, a better understanding of the DDR pathway would help in developing strategies to treat or prevent HCC.

One of the outcomes of activating the DDR is the induction of cellular senescence [7]. Senescence is a process that limits the proliferation of damaged or aging cells in response to multiple types of stress, including DNA damage, telomere shortening and oncogene activation. It is involved in different biological processes, such as carcinogenesis, aging, wound healing, tissue repair and embryogenesis [9]. DNA double-strand breaks, loss of the nuclear lamina protein Lamin B followed by internal and external stresses [10] leads to the appearance of chromatin fragments in the cytoplasm, which eventually activates the cytosolic DNA sensing machine to affect cellular senescence phenotype [11]. During the oncogenesis and development of HCC, cellular senescence acts as a double-edged sword [12]. It prevents the proliferation of damaged cells, thereby preventing tumors from occurring, and also affects the tumor microenvironment by secreting chemokines, proteases and pro-inflammatory cytokines, which are called SASP [8]. DDR defects give rise to genomic instabilities that enhance cancer occurrence and progression through mutation accumulation, unbalancing cellular senescence and apoptosis by inhibiting the cytoplasmic DNA sensing signal pathway. In addition, these defects exhibit targetable vulnerabilities that are relatively specific to cancer cells. For example, DDR inhibitors or regulators can benefit clinical outcome of multiple cancers [13].

DNASE1L3 is a secreted DNase homologous to DNASE1, it is capable of both single- and double-stranded DNA cleavage [14]. It has a unique capacity for digesting membrane-encapsulated DNA, and has a preferential capacity to digest DNA within nucleosomes during apoptosis and necrosis [15]. In combination with DNASE1, it plays a key role in the degradation of neutrophil extracellular traps and cfDNA which in turn reduce organ damage following inflammation [16]. However, differing from its other homologs (DNASE1, DNASE1L1, DNASE1L2), DNASE1L3 contains two functional nuclear localization signals (NLSs) in the mature protein that allows it to shuttle into the nucleus [17]. Thus, whether DNASE1L3 participates in the accumulation of cytoplasmic DNA which in turn affects the progression of HCC via regulating the tumor microenvironments was elucidated.

In this study, we found that downregulated DNASE1L3 expression correlates with poor prognosis of HCC patients. Up-regulated expression of DNASE1L3 relieves cytoplasmic DNA accumulation under DDR activation, in turn cell senescence and SASP were arrested, tumor angiogenesis was impaired. We also found that DNASE1L3 excises these functions through interacting with H2BE. These results indicate DNASE1L3 is a potential biomarker for predicting the prognosis of HCC and identify DNASE1L3 as a regulator of tumor microenvironment via impairing the senescence-associated secretory phenotype in response to stress.

## RESULTS

### DNASE1L3 is downregulated in human HCC tissues and positively correlated with prognosis in resectable HCC

To assess the clinical significance of DNASE1L3 for HCC patients, we first analyzed several publicly available RNA datasets of HCC from The Cancer Genome Atlas (TCGA) and Oncomine database (https://www.oncomine.org). The mRNA expression levels of DNASE1L3 in multiple types of tumors were lower compared with non-tumor tissues. In addition, the expression differences are also significant in multiple HCC cohorts (Supplementary Figure 1A, 1D). Kaplan-Meier curves for the overall survival (OS) and disease-free survival of HCC patients from the TCGA database showed patients with higher mRNA expression level of DNASE1L3 exhibited better prognosis than those with lower expression profile (Supplementary Figure 1B).

**Figure 1:**
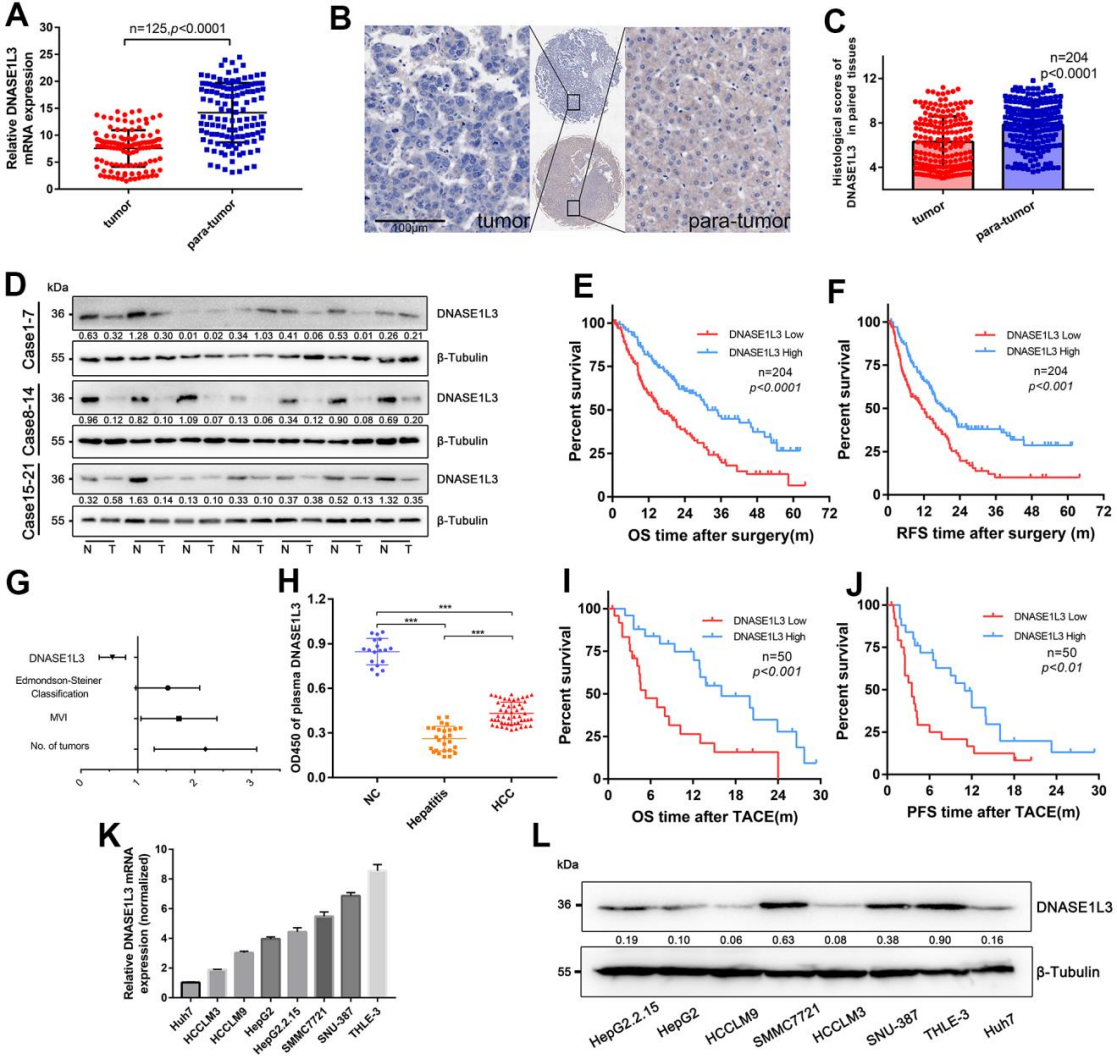
**Downregulated DNASE1L3 is positively correlated with prognosis of resectable or unresectable HCC.** (**A**) The transcriptional level of DNASE1L3 was down-regulated in HCC tissues(n=125) compared to paired para-tumor tissues as tested by RT-qPCR. (**B**) Representative images of IHC staining for DNASE1L3 in HCC and adjacent normal tissues (scale bar, 50 μm). (**C**) The histological scores of DNASE1L3 in 204 paired tissues of HCC was evaluated. (**D**) The translational level of DNASE1L3 between HCC tissues and paired adjacent non-tumor tissues from 21 patients were identified by western blotting. (“T” for tumor, “N” for non-tumor). (**E**, **F**) Kaplan-Meier survival curves of OS and RFS time for 204 patients with HCC. (**G**) Forest plot of risk factors of the OS time using multivariate Cox regression analysis. (**H**) Comparison of plasma levels of DNASE1L3 between patients of HCC(n=50), patients of hepatitis only(n=27) and healthy individuals(n=18). (**I**, **J**) Kaplan-Meier survival curves of OS and PFS time for 50 patients with inoperable HCC. (**K**) RT-qPCR analysis of DNASE1L3 in HCC cell lines and a normal liver cell line (THLE-3). (**L**) Western blot analysis of DNASE1L3 protein expression in HCC cell lines and a normal liver cell line (THLE-3).

To further determine the expression pattern of DNASE1L3 in HCC, we examined the mRNA expression level of DNASE1L3 in 125 paired HCC samples and adjacent non-tumor tissue samples by quantitative real-time PCR (RT-qPCR). The results showed that DNASE1L3 was downregulated in HCC tissues (Figure 1A). To assess the protein expression level of DNASE1L3 in HCC, immunohistochemistry (IHC) analysis was performed using HCC tissue microarray containing 204 paired tumor and para-tumor tissues. The histological scores of DNASE1L3 in paired tissues were evaluated. The results showed the protein expression level was downregulated in HCC tissues (Figure 1B, 1C), consistent with the RT-qPCR results. We then confirmed these findings using Western blotting in another cohort of 21 paired fresh HCC tissues (Figure 1D). These results indicated that the DNASE1L3 protein expression was lower in HCC tissues.

To evaluate the relationship between DNASE1L3 protein expression and clinical pathological features, the cohort of 204 HCC patients subjected to IHC histological scoring was analyzed. Importantly, the results showed that downregulated DNASE1L3 protein expression was significantly associated with advanced tumor size, number of tumors and microvascular invasion (Supplementary Table 1). Kaplan-Meier analyses showed a significant association between low DNASE1L3 expression and poor prognosis of HCC (Figure 1E, 1F). Furthermore, multivariate logistic regression analyses indicated that DNASE1L3 acts as an independent prognostic factor of HCC (Figure 1G and Supplementary Table 2).

### DNASE1L3 was downregulated in the plasmas of HCC patients and was positively correlated with prognosis in unresectable HCC

Since DNASE1L3 is also an extracellular secreted protein [18], furthermore, we analyzed plasma DNASE1L3 protein levels in another cohort of 95 cases encompassing 50 inoperable HCC patients, 27 patients with hepatitis only and 18 healthy individuals. HCC patients exhibited a significantly lower DNASE1L3 levels compared to the normal group, but higher than patients with hepatitis only (Figure 1H). The 50 inoperable HCC patients undergone repeated transcatheter arterial chemoembolization treatment (TACE) and other followed treatments. Kaplan-Meier analyses showed a significant association between low DNASE1L3 expression and poor prognosis in patients with inoperable HCC after TACE (Figure 1I, 1J). We also evaluated the OS and progression-free survival (PFS) time in a cohort of patients treated with sorafenib [19] using KM plotter. The results revealed that low expression levels of DNASE1L3 were associated with lower insensitivity of sorafenib treatment (Supplementary Figure 1C).

Finally, we explored the transcriptional and translational levels of DNASE1L3 in the immortalized normal human hepatic cell line THLE-3 and multiple HCC cell lines, including the SMMC7721, SNU-387, HepG2, HepG2.2.15, HCCLM3 HCCLM9, and Huh7 cell lines. The transcriptional and translational levels of DNASE1L3 found to be suppressed in most HCC cell lines compared to the THLE-3 cell line (Figure 1K, 1L). Taken together, these data implied that the DNASE1L3 protein level is lower in HCC and is closely correlated with poor prognosis of HCC.

### Overexpression of DNASE1L3 relieves cytoplasmic DNA accumulation under DDR activation

**Figure 2:**
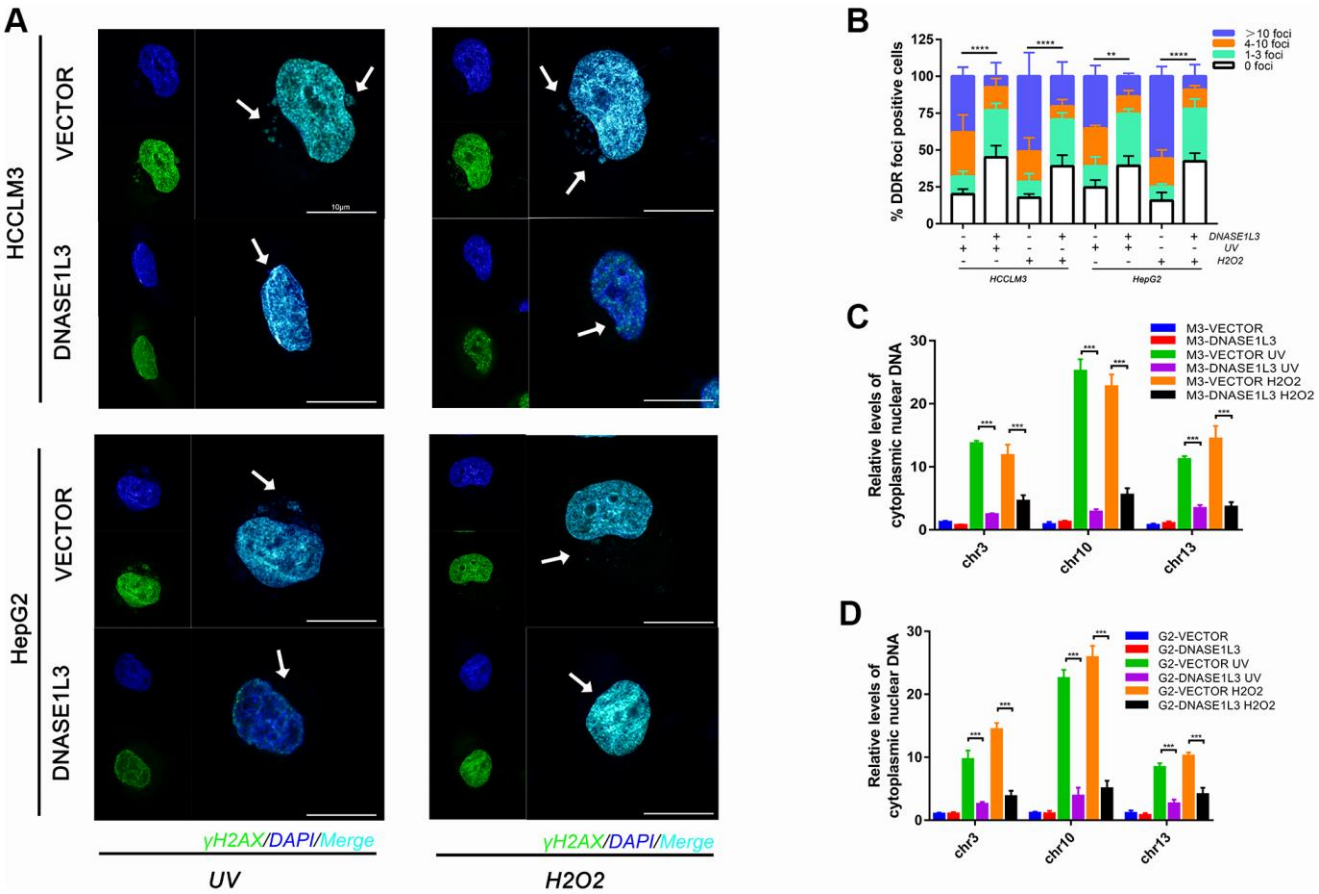
**Overexpression of DNASE1L3 relieves cytoplasmic DNA accumulation under DDR activation.** (**A**) Cytoplasmic accumulation of nuclear DNA in differentially treated cells were assessed, representative images were shown (green, γH2AX; blue, DAPI; Scale bars, 10 μm). (**B**) Quantification of DNA damage foci (DDF). The number of DDF per cell falls into each of the 0, 1-3, 4-10, and >10 counting categories. At least 100 cells counted per group. (**C**, **D**) qPCR analysis of chromosomal DNA in cytoplasmic fraction of cells treated in different group.

Since DDR activation induces cytoplasmic DNA accumulation while the DNase enzymes function as digester of DNA [20], we evaluated the hypothesis that DNASE1L3 may relieve cytoplasmic DNA accumulation under DDR activation. Upon establishing stable DNASE1L3-overexpressing hepatoma cells, DDR was separately induced by UV and H_2_O_2_. Cytoplasmic accumulation of nuclear DNA and DDR were assessed by DNA damage foci (DDF) number (Figure 2A, 2B), reduced DNA synthesis (Supplementary Figure 2A), and qPCR analysis of chromosomal DNA in the cytoplasmic fraction (Figure 2C, 2D). Overexpression of DNASE1L3 inhibited cytoplasmic DNA accumulation while knockdown DNASE1L3 exacerbate the phenotype under DDR activation (Supplementary Figure 3A–3D), at least in certain cultured cell types and such inducing conditions.

### Overexpression of DNASE1L3 relieves cell senescence and SASP under DDR activation

**Figure 3:**
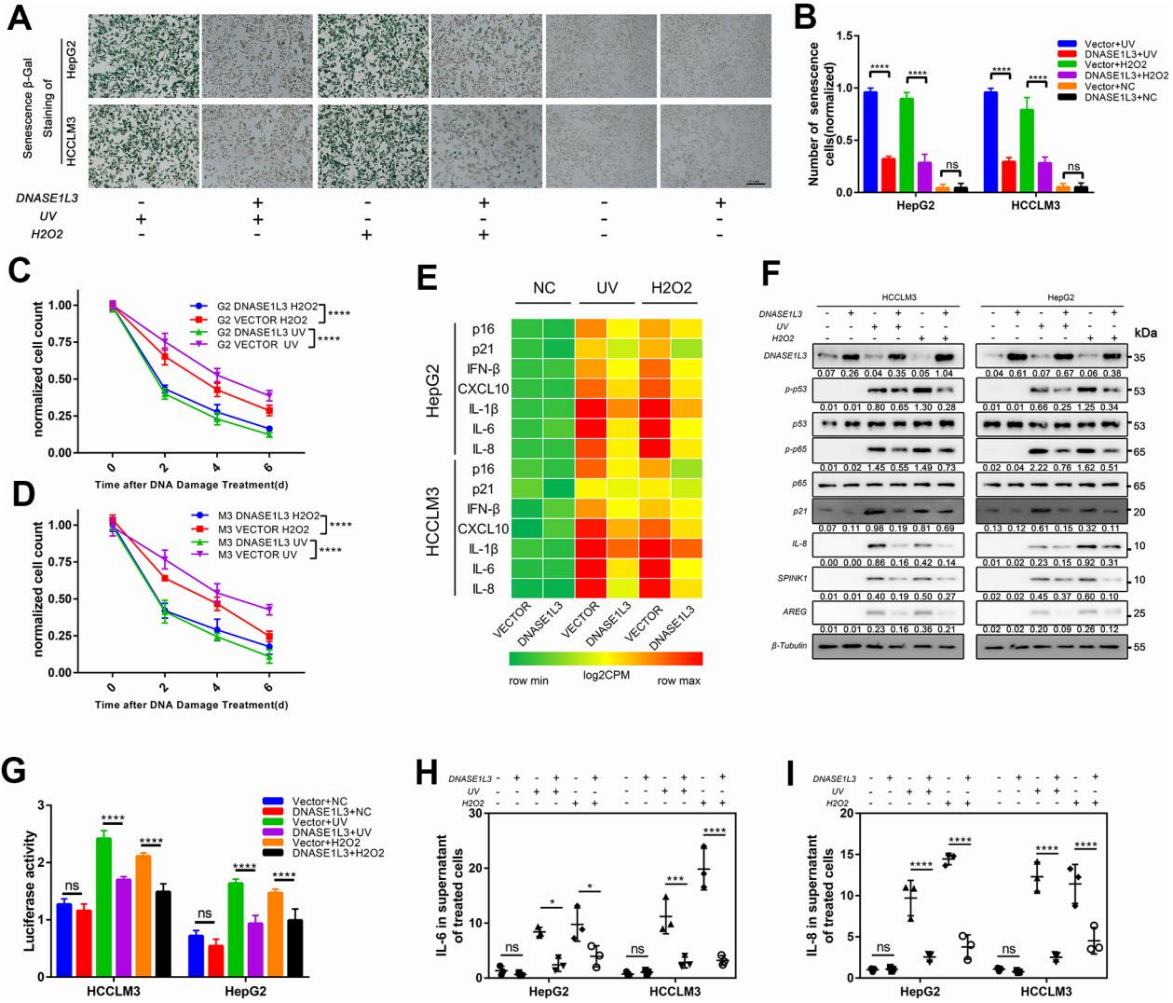
**Overexpression of DNASE1L3 relieves cell senescence and SASP under DDR activation.** (**A**) Representative images of SA-β-Gal staining of cells in differently treated groups (scale bar, 200 μm). (**B**) Statistics of SA-β-Gal staining cells in differently treated groups. (**C**, **D**) Remnant cell number were analyzed by cell counts enumerated from differently treated groups at various time points. (**E**) Transcriptional level of canonical SASP factors in differently treated groups, with values normalized to the vector control per factor. The heatmap for the mean RT-qPCR data is shown. (**F**) Immunoblot analysis of inducible expression change of senescence associated signal pathway and downstream proteins including p53, p65, SPINK1 and AREG in different treated groups. (**G**) Dual luciferase assay showed activated NF-kB signaling in different treated groups. (**H**, **I**) ELISA analysis of IL-6, IL-8 secretion in supernatants from cells in different treated groups.

The prominent concordance between distinctive DNASE1L3 expression and cytoplasmic DNA accumulation under DNA damage response observed in HCC cancer cells prompted us to further explore the functional implications of DNASE1L3 in damaged cells. Notably, UV or H_2_O_2_ treatment induced evident cellular senescence, as indicated by positive SA-β-Gal staining and remarkable morphological alterations. Interestingly, after the induction of DNA damage, both cell lines changed their morphology to resemble a stellate or fusiform morphology with projections, sometimes numerous (Figure 3A). Under DDR activation, DNASE1L3 overexpressed cell lines relieved cell senescence when compared to the vector groups (Figure 3A, 3B). Meanwhile, knockdown of DNASE1L3 made more cells senesced (Supplementary Figure 3F, 3G). However, DNASE1L3 was not shown to affect the senescence ratio without UV or H_2_O_2_ treatment.

Depending on the level of DNA damage, accumulated DNA damage induces cellular apoptosis or senescence [21, 22]. Therefore, we determined whether overexpression of DNASE1L3 could initiate the apoptosis of DNA damaged cells. It was found that the overexpression of DNASE1L3 enhances cell apoptosis in the early stage (day 1to3) after DNA damage (Supplementary Figure 2B). In the late stage (day 7to10) post DNA damage, the remaining cell in the cultures were counted and illustrated in Figure 3C, 3D. The results showed less cells remaining in the culture in DNASE1L3 overexpressed group compared with vector group after DNA damage treatments. Interestingly, even without DNA damage treatments, DNASE1L3 could mildly promotes the apoptosis of HCC cell lines (data not shown here).

Accompanied with the decreased cellular senescence, the expression of IFN-β, a downstream mediator of the cytoplasmic DNA sensing machinery [20], and the other canonical SASP factors such as IL-6, IL-8, CXCL10, SPINK1 and AREG [20, 23] were examined by RT-qPCR, western-blotting or elisa assays (Figure 3E, 3F, 3H, 3I and Supplementary Figure 3E). As supporting evidence, whether the p53-p21 cell arrest signal pathway and NF-κB signal pathway could be impaired by DNASE1L3 was checked by western-blotting and dual luciferase reporter assay. The results obtained from cells in the late stage post DNA damage showed DNASE1L3 suppresses the activation of p53-p21 signal pathway and NF-κB signal pathway under DDR activation (Figure 3F, 3G and Supplementary Figure 3E).

### DNASE1L3 impairs angiogenesis through differential expression of SASP under DDR activation

**Figure 4:**
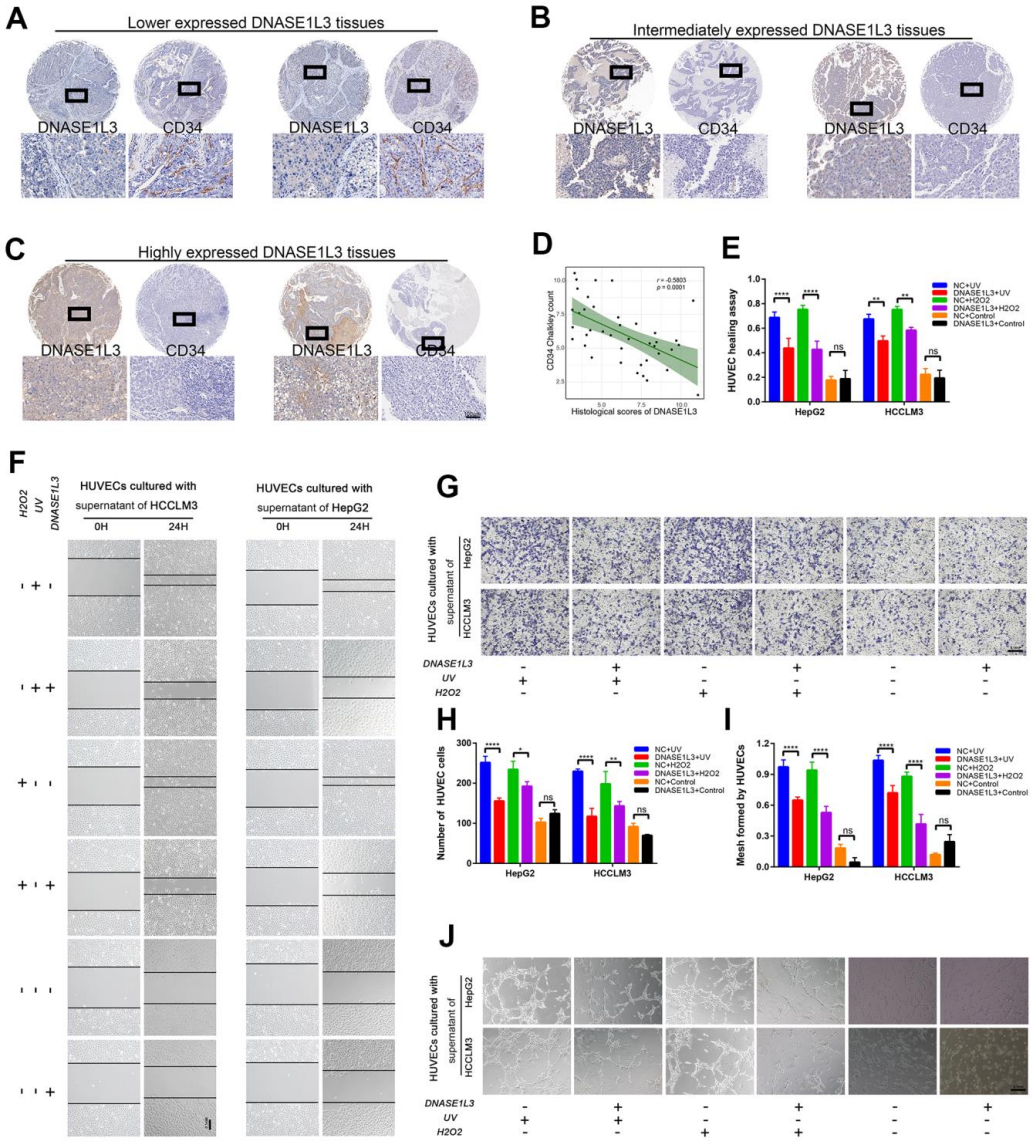
**DNASE1L3 impairs angiogenesis through the differential expression of SASP under DDR activation.** (**A**) Tissue microarray was stained with anti-DNASE1L3 and anti-CD34 antibody, low expression of DNASE1L3 was considerably associated with cancer vasculature invasion. (scale bar, 100 μm). (**B**, **C**) Tumor tissues of DNASE1L3 intermediately or highly expressed were observed to be linked with lower vasculature invasion. (scale bar, 100 μm). (**D**) The histological scores of DNASE1L3 and the CD34 Chalkley counts in HCC tissues was analyzed and showed a significant negative association. (**E**, **F**) The motility of HUVECs were assessed by wound healing assay, the supernatants from cells in differently treated groups were added into the culture of HUVECs, images were taken at 0h and 24h (scale bar, 100 μm). (**G**, **H**) The cellular migration ability of HUVECs were determined by the transwell migration assay. The supernatants from cells in differently treated groups were added into the lower chamber, images were taken after 24h of incubation (scale bar, 100 μm). (**I**, **J**) The tube formation ability of HUVECs were determined by tube formation assay. The supernatants from cells in differently treated groups were added into the culture, images were taken after 6h of incubation (scale bar, 100 μm). The results show the means ± SD from at least three separate experiments.

The expression of DNASE1L3 is associated with the prognosis of HCC with DNA damage treatment or anti-angiogenesis such as TACE or solafenib (Figure 1 and Supplementary Figure 1). Meanwhile, it affects cell senescence and SASP which contain plenty of chemokines, cytokines, and growth factors by cytoplasmic DNA accumulation. We hypothesized that DNASE1L3 affects angiogenesis when DDR is activated. To test this hypothesis, a tissue microarray with 40 HCC samples was used, and the association between DNASE1L3 and CD34 Chalkley count was assessed. Low expression of DNASE1L3 was considerably associated with cancer vasculature invasion (Figure 4A). Tumor tissues of DNASE1L3 intermediately or highly expressed were observed to be linked with lower vasculature invasion (Figure 4B, 4C). The result showed the histological scores of DNASE1L3 was negatively associated with the CD34 Chalkley counts in HCC tissues (Figure 4D). To further confirm the results obtained from the tissue model, we examined HUVEC motility, migration and tube formation using supernatants from cancer cell lines with or without DDR treatments. The results showed, without DDR treatment, the supernatants collected from cell lines overexpressing DNASE1L3 could not initiate the motility, migration and tube formation capacity of HUVEC cells (Figure 4E–4J). However, when DDR was activated with UV or H_2_O_2_ treatments in cancer cells, the angiogenesis of HUVEC cells was enhanced by the supernatants of the treated cells. The opposite phenomenon was observed when knockdown of DNASE1L3 (Supplementary Figure 3H–3K). Accordingly, DNASE1L3 could relives the motility, migration and tube formation ability of HUVEC cells by regulating differential SASP expression of cancer cell lines under DDR activation, at least in certain cultured cell types and such inducing conditions.

### DNASE1L3 interacts with H2BE

**Figure 5:**
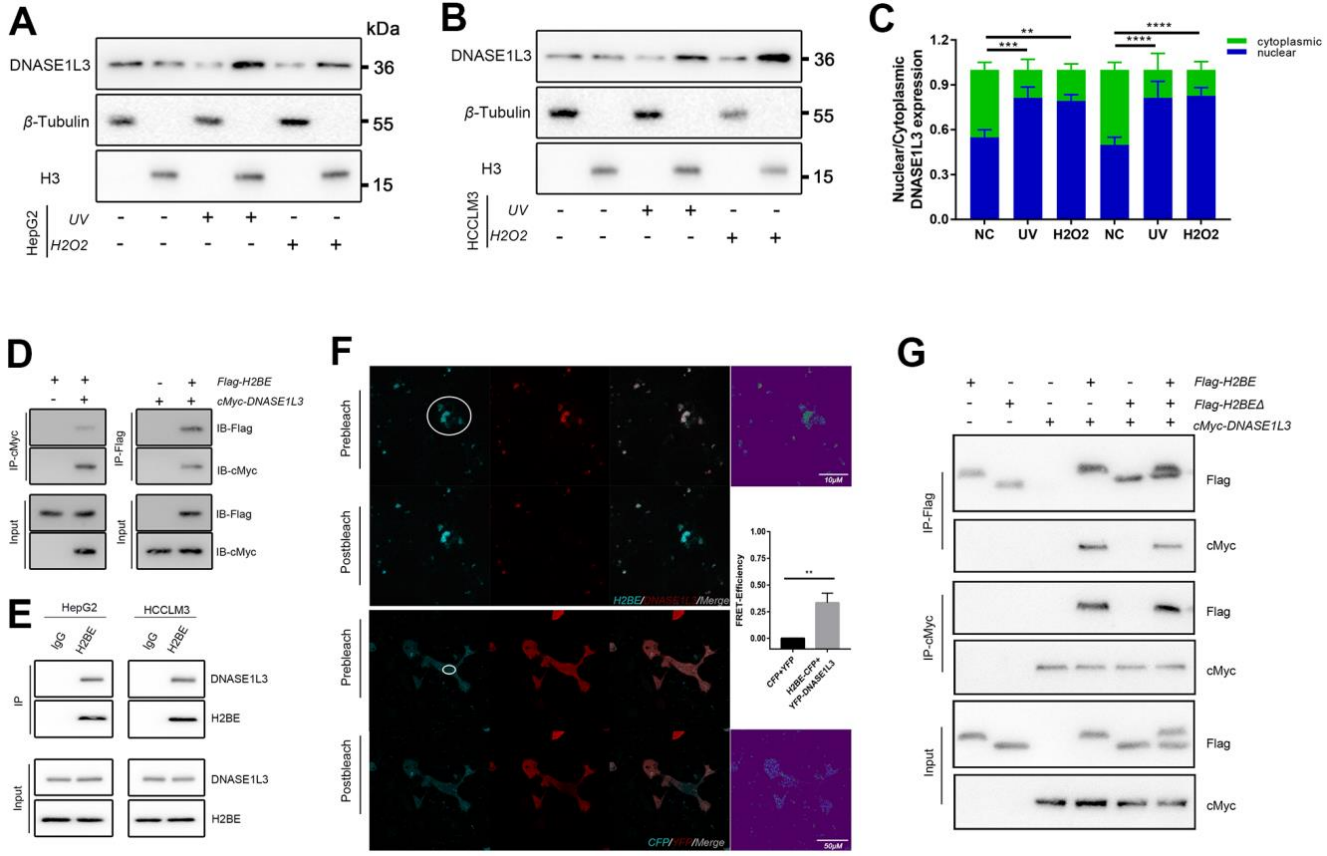
**DNASE1L3 interacts with H2BE.** (**A**, **B**) Immunoblot analysis of DNASE1L3 in the subcellular localization under DNA damage using nucleocytoplasmic separation in two cell lines. (**C**) The result of blotting confirmed that DNASE1L3 translocated to the nucleus in response to DDR activation. (**D**) Lysates of 293T cells overexpressing Flag-H2BE and/or cMyc-DNASE1L3 were subjected to reciprocal co-immunoprecipitation (co-IP) to detect protein interaction. (**E**) HepG2 and HCCLM3 cell lysates were subjected to co-IP and immunoblot to detect endogenous H2BE and DNASE1L3 interaction. (**F**) FRET assay for DNASE1L3-H2BE interactions in living cells. FRET efficiency were calculated by Leica TCS SP8 software (FRET_eff_= (D_post_-D_pre_)/D_post_). White circles identify FRET area. (**G**) Co-IP of cMyc-DNASE1L3 and Flag-tagged H2BE or H2BEΔ with N-terminal region (amino acids 1-28) deleted.

DNASE1L3 was reported possessed two nuclear localization signal peptides [24]. Meanwhile, VP-16, a topoisomerase inhibitor interferes with the action of topoisomerase enzymes (topoisomerase I and II), in turn causes single-strand and double-strand breaks in DNA and produces cytotoxicity, could facilitate DNASE1L3 translocation to the nucleus [25]. We identified DNASE1L3 relieved the cytoplasmic DNA accumulation under DDR activation (Figure 2). Thus, we assume that DNASE1L3 may digest the damaged DNA fractions via enhancing translocation to the nucleus. In order to further validate this conclusion, we performed the nucleocytoplasmic separation followed by western blot experiments, using cell lines with or without UV or H_2_O_2_ treatments. The results showed the translocation to the nucleus of DNASE1L3 was enhanced upon DDR activation (Figure 5A–5C).

At this point, the mechanism of how DNASE1L3 finish the digest of damaged DNA fractions puzzled us. We analyzed IntAct Molecular Interaction Database (https://www.ebi.ac.uk/intact/interaction/EBI-20919708) and checked coIP-MS results in our previous experiment(unpublished). The DSSO crosslink assay showed DNASE1L3 could bind to H2BE [26], previous coIP-MS results (Supplementary Figure 4A) showed DNASE1L3 have connections with multiple histones (H3, H2AJ, H2A1C, etc.). All these findings indicate that DNASE1L3 may exert its functions by binding H2BE. To validate the physical interactions between DNASE1L3 and H2BE, we enforced the expression of Flag-H2BE and cMyc-DNASE1L3 in 293T cells for reciprocal immunoprecipitation and confirmed associations between the two proteins (Figure 5D). Moreover, co-immunoprecipitation using HepG2 and HCCLM3 cell lysates validated the specific interaction between endogenous DNASE1L3 and H2BE in HCC cells (Figure 5E). To substantiate this binding preference, we then used a fluorescence resonance energy transfer (FRET) assay to assess the relative proximity of DNASE1L3 and H2BE in live cells. In HepG2 cells, co-expression of YFP-DNASE1L3 and H2BE-CFP produced FRET, which demonstrated an association between DNASE1L3 and H2BE (Figure 5F).

To define the precise region of H2BE DNASE1L3 binding with for this interaction, we enforced the full-length cMyc-tagged DNASE1L3 in combination with Flag-tagged respective fragments of H2BE in HepG2 cells (Figure 5G). The N-terminal region of H2BE (amino acids 1-28) is the area on the protein surface, and it is not winded by DNA sequence (Supplementary Figure 4B). We deleted this domain and reconstructed a H2BEΔ (Supplementary Figure 4C). Interestingly, the truncated protein lost the ability to interact with DNASE1L3 (Figure 5G), suggesting that the interacting module likely resides in amino acids 1-28.

### DNASE1L3 impairs angiogenesis by interacting with H2BE

**Figure 6:**
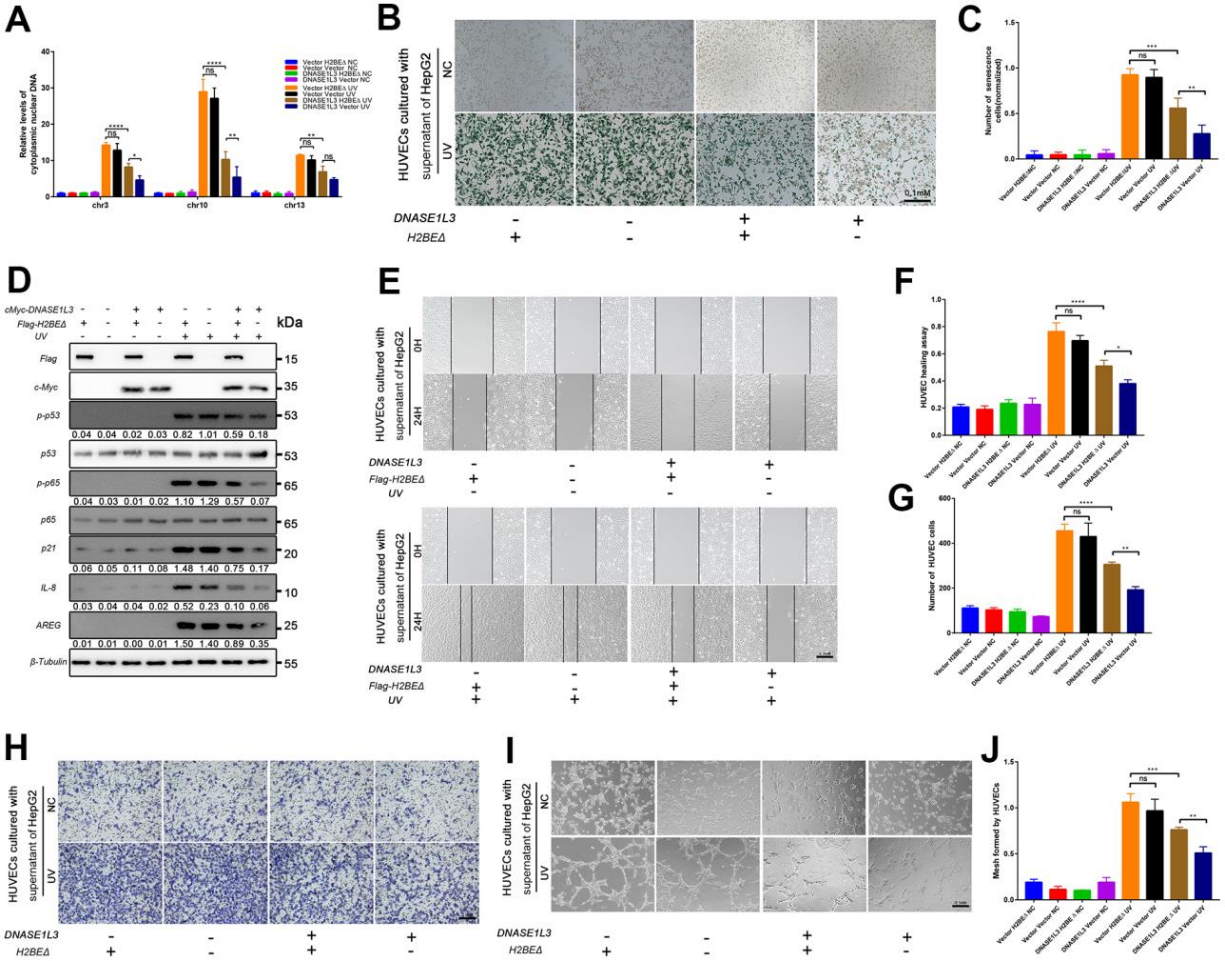
**DNASE1L3 impairs angiogenesis by interacting with H2BE.** (**A**) qPCR analysis of chromosomal DNA in the cytoplasmic fraction of differently treated HepG2 cells. (**B**) Representative images of SA-β-Gal staining of cells in differently treated groups (scale bar, 100 μm). (**C**) Statistics of SA-β-Gal staining cells in differently treated groups. (**D**) Immunoblot analysis of inducible expression change of senescence associated signal pathway and downstream proteins including p53, p65, SPINK1 and AREG in differently treated groups. (**E**, **F**) The motility of HUVECs were assessed by wound healing assay, the supernatants from cells in different treated groups were added into the culture of HUVECs, images were taken at 0h and 24h (scale bar, 100 μm). (**G**, **H**) The cellular migration ability of HUVECs were determined by the transwell migration assay. Cell supernatants from in differently treated groups were added into the lower chamber, images were taken after 24h of incubation (scale bar, 100 μm). (**I**, **J**) The tube formation ability of HUVECs were determined by tube formation assay. Cell supernatants from differently treated groups were added into the culture, images were taken after 6h of incubation (scale bar, 100 μm). The results show the means ± SD from at least three separate experiments.

All of our analyses described thus far suggest that the N-terminal region of H2BE may contribute to the function of DNASE1L3 excised in SASP associated angiogenesis under DDR activation. To test this idea, we next sought to determine the rescue experiments using the truncated protein, Flag-tagged H2BEΔ. Interestingly, we noted that the cytoplasmic DNA accumulation increased when co-transfect H2BEΔ with DNASE1L3 compared with vector and DNASE1L3 (Figure 6A). Cell senescence was also enhanced in the following SA-β-Gal staining assay (Figure 6B, 6C). We also assessed p53-p21 and NF-κB signaling pathways under DDR activation, without the N-terminal region of H2BE, the function of DNASE1L3 was restricted (Figure 6D). We continued to validate its impact on angiogenesis under DDR activation. HUVEC motility, migration and tube formation assays using supernatants from H2BEΔ co-transfected HepG2 cells with or without DDR treatments were performed (Figure 6E–6J). The results strongly suggest the involvement of both the N-terminal region of H2BE and DNASE1L3 in tumor angiogenesis through regulating senescence-associated secretory phenotype in response to stress.

### DNASE1L3 arrests tumor angiogenesis via regulating senescence-associated secretory phenotype in response to stress

**Figure 7:**
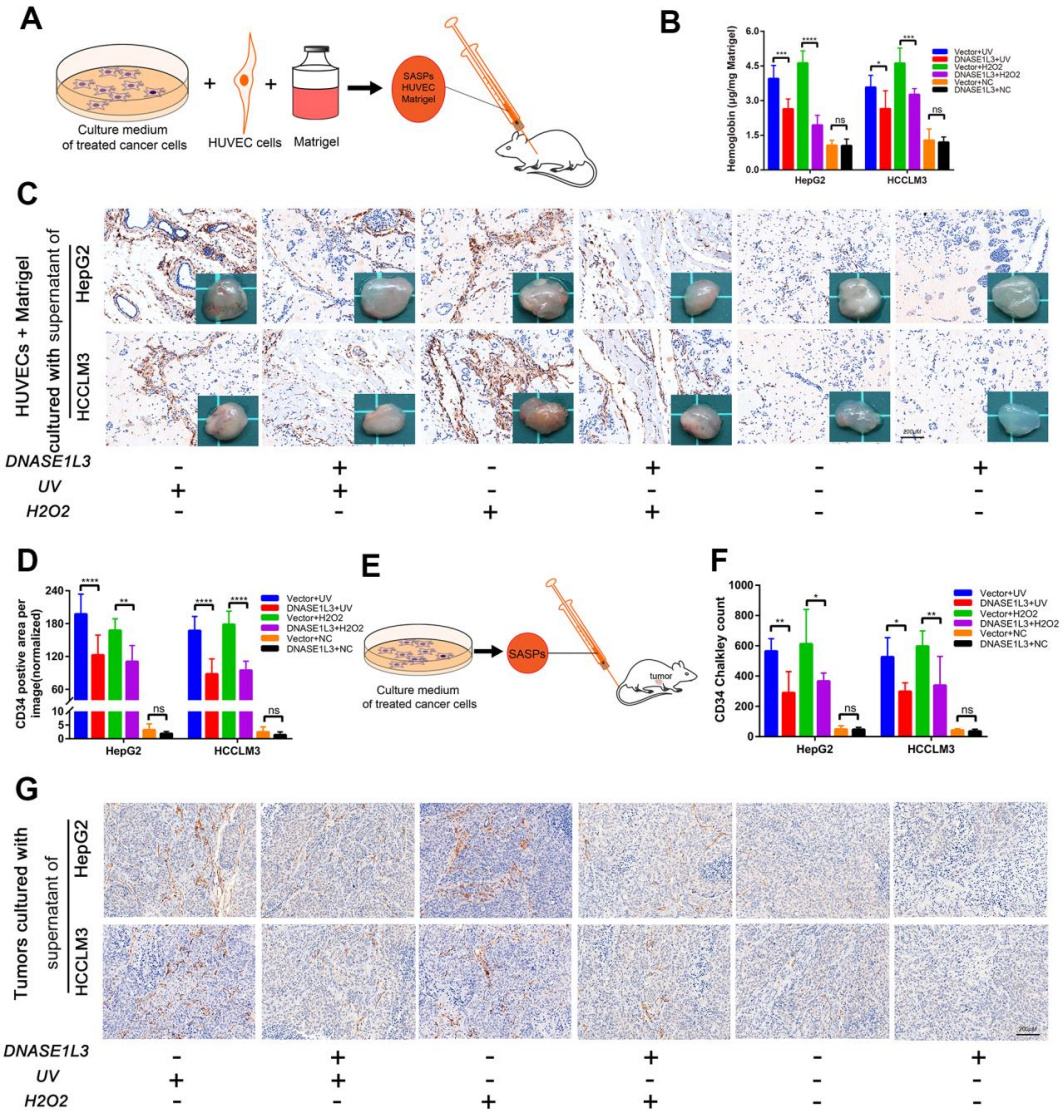
**DNASE1L3 arrests tumor angiogenesis by regulating the senescence-associated secretory phenotype in response to stress.** (**A**) Matrigel plugs supplemented with cell supernatants from differently treated groups were subcutaneously injected into mice. (**B**) Hemoglobin content was assessed at day 10 after injection. (**C**) Representative images of CD34 staining of the matrigel plugs in differently treated groups (scale bar, 200 μm). Gross view observation of the matrigel plugs were shown in the bottom right corner of each figure. (**D**) CD34 positive area was evaluated in each group. (**E**) Constructed subcutaneous tumor model was used to investigate the role of DNASE1L3 in tumor angiogenesis. (**F**) Vascular density was quantified by CD34 Chalkley count under IHC. (**G**) Representative images of CD34 staining of subcutaneous tumors in differently treated groups (scale bar, 200 μm).

The matrigel plug assay was used to assess the ability of DNASE1L3 to inhibit angiogenesis *in vivo*. Matrigel plugs supplemented with supernatants of genetically edited cells with or without DNA damage treatments were subcutaneously injected into mice (Figure 7A). The plugs were then excised, photographed, and assessed for hemoglobin content and CD34 positive area 10 days after injection. Angiogenesis levels within the plugs were evaluated by the degree of hemoglobin content. The plugs were sliced and CD34 positive areas were evaluated. The matrigel supplemented with the supernatant of DNASE1L3 overexpressed cells treated by UV or H_2_O_2_ exhibited a lighter color depth compared to the vector group, but deeper than cells without DNA damage treatment (Figure 7B). The observed difference in the tint was confirmed by quantification of hemoglobin and CD34 positive areas (Figure 7C, 7D).

Finally, we further established a subcutaneous tumor model to investigate the role of DNASE1L3 in tumor angiogenesis. Mice were inoculated with Hepa1-6 cells first, then supernatants of different groups of cells treated by UV or H_2_O_2_ treatments were injected through the tail vein, twice per week, for 2 weeks for a total of four doses (Figure 7E). Vascular density was quantified by CD34 Chalkley count under IHC. The results show DNASE1L3 arrests tumor angiogenesis through the secretions from cells treated with DDR activation (Figure 7F, 7G). These *in vivo* results, coupled with the *in vitro* and *ex vivo* observations, strongly suggest that DNASE1L3 is a suppressor of angiogenesis.

## DISCUSSION

**Figure 8:**
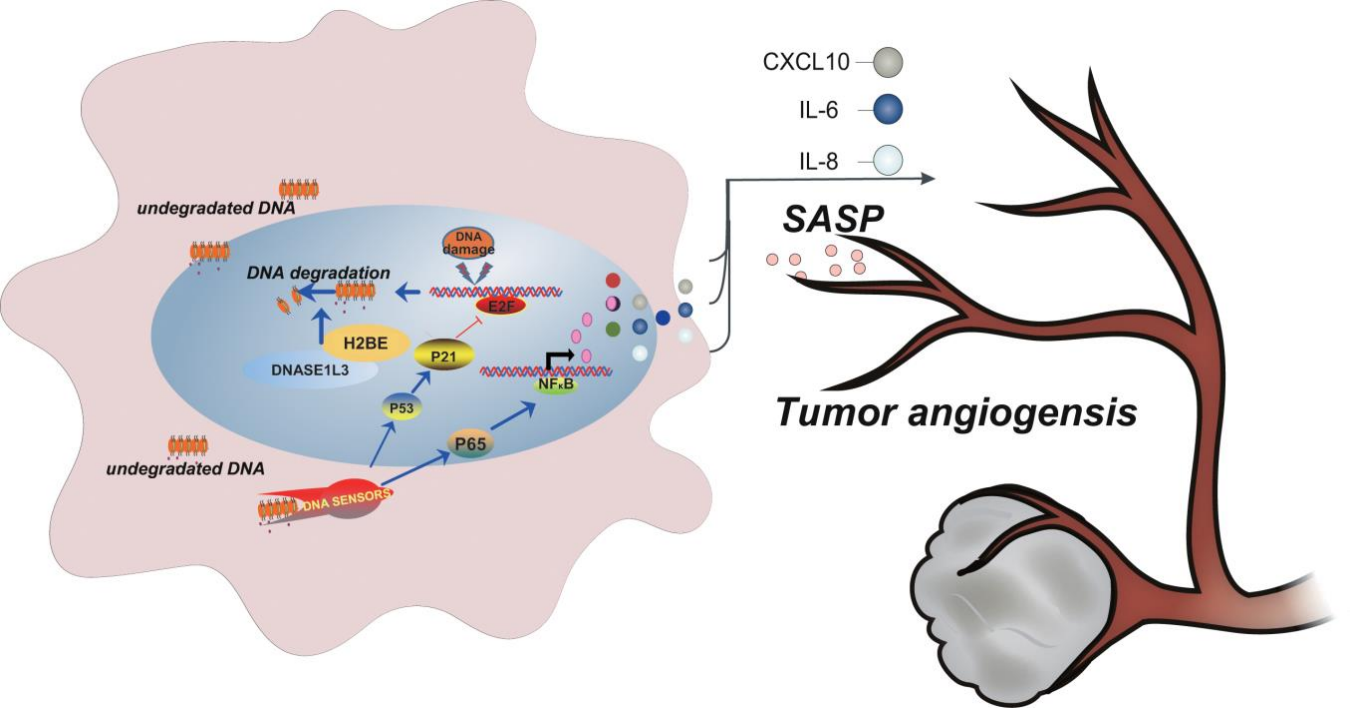
**Model depicting DNASE1L3 regulation of tumor angiogenesis by controlling the senescence-associated secretory phenotype in response to stress.**

Our findings from the present study fill some gaps between the poor prognosis of HCC and the low expression of DNASE1L3. Our data indicates that DNASE1L3 excises the function in dealing with the accumulated cytoplasmic DNA of nuclear origin provoked by DNA damage. This event results in the relief of DNA damage response, in turn down regulates the occurrence of cellular senescence which affects the tumor angiogenesis through senescence-associated secretions into the microenvironment (see model in Figure 8).

DNASE1L3 is distributed in the endoplasmic reticulum, cytosol, nucleus and extracellular space [16, 27]. It translocates to the nucleus upon cleavage of its endoplasmic reticulum-targeting motif during apoptosis [28, 29]. In our experiments, DNASE1L3 translocates from the cytoplasm to the nucleus in response to UV or H_2_O_2_ induced DNA damage. As an intracellular endonuclease, it has been investigated that DNASE1L3 associates with DFFB to digest DNA and fragment it into internucleosomal repeats of 153-200 bp [18, 30–32]. The DFFB is involved in the initial cleavage of DNA into 50kb fragments, but not sufficient for acetaminophen-induced internucleosomal DNA fragmentation [32]. Cells deficient in DFF45 lack the ability to generate such DNA fragments in response to various inducers of apoptosis, but have the capacity of resist apoptotic inducers [25]. DNASE1L3 mediates acetaminophen-induced internucleosomal DNA fragmentation [33]. In this study, overexpression of DNASE1L3 enhanced the apoptosis of DNA damaged HCC cell lines. This indicates that, defects in DNASE1L3 enhances the accumulation of cytoplasmic DNA and triggers the activation of DNA sensors. However, it maintains the homeostasis through anti-apoptosis.

Evolving eukaryotes developed the ability to maintain homeostasis not just in cells but in the whole organisms too. Homeostatic maintenance leads to a powerful rewiring of adaptive stress responses [34, 35]. Responses to the disturbance of intracellular or extracellular microenvironments, the cells and the body attempt to restore the physiological functions. However, if stress adaptation fails, cells exhibit one of two ultimate destinies: i. cell senescence, corresponding to their irreversible proliferation and inactivation [35, 36]; or ii. regulation of cell death (RCD), through one of a variety of non-exclusive and highly interrelated pathways [35, 37].

When cellular fitness is irreparably damaged, the dynamic balance between senescence and RCD are used to maintain body homeostasis. However, the outcomes vary and are determined by signal characteristics, spatiotemporal parameters, and cellular capacity to respond [9, 38]. In cases of minor disruptions of cellular processes, brought about by low levels of stress, damage is reversed and the structural and functional integrity of cells restored. Alternatively, damage can be irreversible, causing tissue degeneration and cell death. Between both situations, cells acquire a non-proliferative but viable state, distinct from G0 quiescence and terminal differentiation, with permanent structural and functional changes, termed cellular senescence [9]. But one thing still needs to be pointed out, either the cells undergoing successful (recovery of cellular homeostasis) or unsuccessful (senescence or RCD) responses to stress, they communicate their status to the microenvironment, ultimately affecting organismal adaptation [34].

In our research, cancer cells defected of DNASE1L3 tend to avoid cell death by surpassing apoptotic processes. In contrast, overexpression of DNASE1L3 provokes more cellular apoptosis than senescence during DNA damage. This must be relevant to its function in processing DNA fragmentation from ~50kb into mononucleosome of 153 bp [18]. Low expression of DNASE1L3 decrease the degradation efficiency of unsuccessful repaired DNA to some extent. Thus, in DNASE1L3 relieves the activation of p53-p21 signal pathway and NF-κB pathway via repressing cytoplasmic DNA accumulation during DNA damage. Meanwhile, the DNASE1L3-dependent DNA fragments also provoke the expression of numerous SASP factors and promote tumor angiogenesis. Interestingly, in our retrospective clinical studies of HCC, the reduced DNASE1L3 expression is associated with not only less favorable outcome after hepatectomy, but also the poor prognosis of DNA damage therapy (ischemia and hypoxia, accompanied by oxidative stress and mitochondrial dysfunction induced by TACE [39]) and anti-angiogenic therapy (Sorafenib).

In summary, this study, for the first time, confirms that DNASE1L3 affects the angiogenesis via regulating the expression of numerous SASP factors in response to stress. Mechanistically, during DNA damage, DNASE1L3 translocates to the nucleus, interacts with H2BE, participates in the degradation of unsuccessful repaired DNA, and relieves tumor cell senescence as well as the expression of SASP factors contributed to the microenvironments. Based on these findings, we propose that DNASE1L3 may act as a vital predictive biomarker for associated clinical DNA damage treatments.

## MATERIALS AND METHODS

### DNA damage treatment and induction of senescence

For ultraviolet (UV) radiation-treatment, approximately 2 × 10^5^ cells was cultured in each well of a 6-well plate. When DNA damage performed, the medium was taken off, cells were irradiated with UVB, 20-30 mJ/cm^2^. Then, incubate the cells in 2 mL of DMEM with 10%FBS for another 7 days. For oxidative stress treatment, approximately 2 × 10^5^ cells were cultured and incubated in each well of a 6-well plate. A solution of ~150 μM hydrogen peroxide in complete cell culture was prepared by adding 75 μL of 30% hydrogen peroxide in 50 mL of complete cell culture. When DNA damage performed, the medium of the cells was aspirated and washed once with fresh PBS. Then, 2 mL of DMEM with hydrogen peroxide was added, and incubated for 3 days. The preceding step was repeat twice, and the cells were incubated for another 6 days.

### SA-β-Gal staining

SA-β-Gal staining was processed with Senescence β-Galactosidase Staining Kit (Beyotime, China), following the manufacturer’s recommendations. Briefly, cells with or without DNA damage treatments were cultured for 7 days. After that, the samples were fixed for 15 min followed by washing the plate or slides with PBS for 3 times. Next, the plates were rinsed with PBS, stained with β-galactosidase staining solution, incubated overnight at 37° C in a dry incubator without CO_2_. Then, the slides were rinsed with tap water. Finally, we analyzed the blue staining under a fluorescence microscope.

### Fluorescence resonance energy transfer (FRET) assay

HepG2 cells plated on poly-l-lysine–coated glass-bottomed dishes (Beyotime) were cotransfected with reconstructed pEYFP-C1 and pECFP-N1 plasmid. The basic vectors (pEYFP-C1 and pECFP-N1) were used in the control experiments. After cultured for 96 h, they were treated with UV radiation and cultured for another 8 h. Samples were imaged using Leica TCS SP8 confocal microscope equipped with three photomultipliers (PMTs). The laser was tuned to lines 458 and 514 to excite CFP (458 nm) and YFP (514 nm). With a META spectral detector, emission profiles were generated by scanning emission spectra of H2BE-CFP and DNASE1L3-YFP across a series of wavelengths (462 to 633 nm). FRET was measured using acceptor photo-bleaching according to the method reported previously [40]. The FRET efficiency were calculated by Leica TCS SP8 software (FRET_eff_= (D_post_-D_pre_)/D_pos_).

### Ethics approval and consent to participate

Ethical approval was obtained from Ethics Committee of Zhongnan Hospital of Wuhan University, and written informed consent was obtained from each patient.

### Consent for publication

All authors have agreed to publish this manuscript.

### Availability of data and materials

The datasets used and analysed during the current study are available from the corresponding author on reasonable request.

## Supplementary Material

Supplementary Methods

Supplementary Figures

Supplementary Tables
