# Supplementary material for: DNASE1L3 arrests tumor angiogenesis by impairing the senescence-associated secretory phenotype in response to stress
Source: Aging (Albany NY). 2021 Mar 19;13(7):9874–99. doi: 10.18632/aging.202740 (PMC8064203; doi:10.18632/aging.202740)
Supplement: Supplementary Methods [file aging-13-202740-s001.pdf]

SUPPLEMENTARY MATERIALS

Supplementary Methods

Ethical application

Use of human subjects

Written informed consent was given by all participating patients or their legal representatives. Ethics committee approval was obtained from the ethics committee of Zhongnan Hospital.

Use of experimental animals

All animal experiments were approved by the Wuhan University Animal Welfare and Ethical Review Body (AWERB) and the study was approved by the home office. All animal care and experimental procedures were in accordance with Roche guidelines and national/international guidelines for animal care.

Cell lines and culture

The human hepatocellular carcinoma cell line HepG2, SMMC7721, SNU-387, HepG2.2.15, HCCLM3, HCCLM9, Huh7 and mouse hepatocellular carcinoma cell line Hepa1-6 as well as HEK293T and THLE-3 were all grown in DMEM (HyClone, USA) supplemented with 10% fetal bovine serum(FBS) (HyClone, USA). Cells were cultured in monolayers in 75 cm^2 or 10 cm culture dishes humidified atmosphere containing 5% CO_2.

Cell culture supernatant concentration and extraction

The supernatants of cells in different groups treated with or without UV or H2O2 after 7days were collected and centrifuged at 1,000 g × 10 minutes. After that, the centrifuged supernatant was filtered by Amicon Ultra filter device (Millipore, USA), centrifuged at 4,500 g × 30 minutes. To this point, concentrated protein-containing liquid was obtained.

Immunofluorescence and immunohistochemistry (IHC)

HCC specimens were obtained from 204 patients (aged from 18-80 y) accepted curative liver resection operation in Zhongnan Hospital of Wuhan University in Wuhan, China. No patients underwent preoperative anti-cancer treatment. For Immunofluorescence analysis, cells grown on coverslips were treated according to previous method [1]. For immunohistochemical analysis, formalin-fixed paraffin embedded tissue microarray slides from HCC patients were probed with DNASE1L3 antibody (ab203669, Abcam). Formalin-fixed paraffin-embedded samples were also obtained from xenograft tumors and then probed with antibodies against CD34 (ab81289, Abcam). After that, positive cells were identified with DAB+ as a chromogen. To analyze the expression level of DNASE1L3 and CD34, slides were scanned by Aperio ScanScope (Aperio) scored modifying a method as previously reported [1].

Plasmid constructions

Full-length sequence of DNASE1L3 with a C-terminal Myc tag and H2BE with a N-terminal Flag tag was cloned into pcDNA3.1(-) separately. Deletion mutants of H2BE, H2BEΔ29-126 with a N-terminal Flag tag were constructed by PCR and cloned into pcDNA3.1(-). For the FRET assay, the full-length sequence of DNASE1L3 and H2BE were cloned into pEYFP-C1 and pECFP-N1 separately. For shRNA experiments, oligonucleotides targeting homo sapiens DNASE1L3(1# 5′- GGCTTGG AAGAAACACATA -3′; 2# 5′- GCATAACGTACAA CTATGT -3′), were annealed and cloned into the pENTR/U6 shRNA vector.

Quantitative real-time PCR

Total RNA was extracted from cultured cells and tissues by TRIzol reagent (Invitrogen) and reverse transcribed into cDNA by HiScript® III 1st Strand cDNA Synthesis Kit (+gDNA wiper) (Vazyme). RT-PCR was performed using ChamQTMSYBR®qPCR Master Mix(High ROX Premixed) (Vazyme). All PCR primers are summarized in Supplementary Table 3.

Isolation of cytosol DNA fractions

Cytoplasmic DNA was extracted according to a previously reported method [2]. Briefly, cells were centrifuged at 200g × 5 min, suspended and homogenized in 0.3 M sucrose buffer, overlaid on 1.5 M sucrose buffer, centrifuged at 18,506 g ×10 min,purified by 0.4 mg/ml Proteinase K (Beyotime) treatment, phenol/chloroform extraction and ethanol precipitation with a carrier (Dr. GenTLE® Precipitation Carrier, Takara BioInc.). RT-qPCR was used to assess the amount of cytoplasmic DNA [2]. The PCR primers for human chromosome 3, 10, and 13 are shown in Supplementary Table 3.

Western blot analysis

Cells or tumor tissues were lysed in RIPA lysis buffer with 1% Protease inhibitor cocktail (Beyotime, China). The protein concentration was obtained by bicinchoninic acid (BCA) assay (Beyotime, China). Then the samples were separated by SDS-PAGE and transferred onto a PVDF membrane (Millipore, USA). Membranes were

probed with appropriate dilutions of specific primary antibodies overnight at 4° C, then HRP-conjugated secondary antibodies were used for further detection. The primary antibodies used in this assay are listed in Supplementary Table 4.

ELISA assay

Cells were counted for normalization followed by cell culture supernatant collected as previously illustrated. Human IL-6, IL-8 immunoassay was tested using kits per the manufacturers instructions. (Proteintech, China).

Coimmunoprecipitation

To evaluate the interaction between DNASE1L3 and H2BE, plasmids Flag-H2BE and cMyc-DNASE1L3 were cotransfected into HEK 293T cells. Lysates of HepG2, HCCLM3, HEK293T cells were pre-cleaned and then coimmunoprecipitation was performed with the Pierce Co-Immunoprecipitation kit (Thermo Scientific). The lysates in IP lysis buffer were incubated with anti-Flag, anti-Myc, anti-H2BE antibodies or IgG overnight at 4° C, then the proteins were eluted in 60 μl and separated by western blotting.

NF-κB reporter assays.

NF-κB transcription activity was identified by luciferase signals derived from a pNF-kB-TA-Luc construct that encodes multiple copies of NF-κB binding sequences. pGL4.70(hRluc) plasmid was co-transfected for transfection efficiency controls. Luciferase activities of cell lysates were tested using a luminometer and Dual Luciferase Reporter Gene Assay Kit (Beyotime), according to the manufacturer's instructions.

Flow cytometric analysis

HepG2 and HCCLM9 cells transfected with vector or DNASE1L3 were treated with or without H2O2 or UV treatments. After that, cells were continued cultured for another 24 hours and harvested. Samples were stained with an annexin V-FITC/PI apoptosis kit (MultiSciences). The cells were then analyzed by flow cytometry to identify apoptosis (Beckman Coulter).

5-Ethynyl-2′-deoxyuridine (EdU) incorporation assay

EdU incorporation assay was operated using the BeyoClick™ EdU-594 imaging detection kit (Beyotime, China). HepG2 and HCCLM9 cells transfected with vector or DNASE1L3 treated with H2O2 or UV treatments were cultured for another 24 hours after DNA damage treatments, then EdU labelling medium was added and incubated for 24 hours. The cells were proceeding as the manufacturer's instructions recommended.

Wound healing assay

HUVECs were suspended in DMEM with 10% FBS, and seeded at a density of 500,000 cells per well in 6-well culture plates. Upon reaching 100% confluence, wounds were made using a 200 μl plastic pipette tip. The medium was then changed to DMEM mixed with concentrated supernatant from different groups and incubated for further tests. Photos were recorded at 0 and 24 h.

Cell migration assay

Cell migration assays were performed using transwell chambers (8-μm pore size; Corning) in 24 well plates. In the lower chamber, concentrated cell culture supernatants from different groups were mixed in complete culture separately; in the upper chamber, approximately 50,000 HUVECs suspended in DMEM were added. After incubated for another 24 hours, the cells in the upper chamber were fixed with methanol and stained with 0.1% crystal violet. Cells on the upper side of the transwell membrane were wiped off with a cotton swab, cells on the underside of the transwell membrane were photographed and quantified under a microscope.

Tube formation assay

200 μl of Matrigel (Corning® Matrigel® Matrix, USA) was added to each well of a 24-well plate. HUVECs were suspended in DMEM mixed with concentrated cell culture supernatant from different groups and seeded onto the Matrigel at the density of 200,000 cells per well followed by the Matrigel polymerized in the incubator for 60 min. Then, the cells were incubated for 6 h, tubes were visualized under a microscope and the meshes formed by HUVECs were quantified.

Subcellular fractionation assay

The separation of nucleus-cytoplasm in HepG2 and HCCLM3 cells were performed following the manufacturer's instructions of the PARIS Kit (Invitrogen). β-Tublin and H3 were employed as the indicators for quantification of the fractions.

Matrigel plug assay

To evaluate the angiogenic response *in vivo*, matrigel growth factor reduced (0.40 mL, Corning) mixed with heparin (10 U/mL), a total of 10^6 HUVECs and concentrated cell culture supernatant (0.1mL) from

different groups were mixed separately and subcutaneously injected into the flanks of 6-week-old BALB/c nude mice (Vital River, Beijing, China). On day 10 post injection, matrigel plugs were removed and sliced into two parts, hemoglobin quantities were then evaluated. The other parts of matrigel plugs were fixed in formalin and embedded with paraffin for the followed IHC assays.

In vivo assay

For tumor angiogenesis assay *in vivo*, Hepa1-6 cells were subcutaneously injected into 5-week-old male BALB/c nude mice (Vital River, Beijing, China) to induce tumor formations. The model was established after feeding for a week. Then, concentrated cell culture supernatants from different groups were separately injected via tail vein for the subsequent two weeks (twice per week, for 2 weeks for a total of four doses). Mice were sacrificed at 8 weeks. The vascular density was quantified by CD34 Chalkley count under IHC.

Bioinformatics data analysis

For TCGA analyses, RNA sequencing datasets were obtained from cBioportal (www.cbioportal.org). The expression data of DNASE1L3 were log2TPM transformed and compared between tumors and normal tissues. The OS and RFS time results were obtained from GEPIA web server [3]. For the cohort of HCC patients treated with sorafenib, a total of 364 cases were enrolled and 30 patients who had been subjected to sorafenib treatment were selected. Liver cancer RNA-seq data, OS time and PFS time were analyzed using KM plotter [4].

Statistical analysis

Data were shown as means±SD or as a percentage from at least three independent experiments. IBM SPSS statistical software (IBM Corporation, USA) and GraphPad Prism (GraphPad Software, USA) were used to perform the statistical analysis. The relationship between DNASE1L3 expression and clinicopathological characteristics was determined using chi-Square test. Differences between experimental groups were assessed by Student's t-test or one-way ANOVA. Survival time was calculated by Kaplan-Meier and log-rank tests. Independent predictors of OS time were detected using Cox regression analysis (the criteria for the entry into the logistic regression model was $P < 0.05$). Statistical significance was set at $^{*}P < 0.05$, $^{**}P < 0.01$, $^{***}P < 0.001$.

REFERENCES

1. Ma Z, Guo D, Wang Q, Liu P, Xiao Y, Wu P, Wang Y, Chen B, Liu Z, Liu Q. Lgr5-mediated p53 repression through PDCD5 leads to doxorubicin resistance in hepatocellular carcinoma. Theranostics. 2019; 9:2967–83. https://doi.org/10.7150/thno.30562 PMID:31244936

2. Takahashi A, Loo TM, Okada R, Kamachi F, Watanabe Y, Wakita M, Watanabe S, Kawamoto S, Miyata K, Barber GN, Ohtani N, Hara E. Downregulation of cytoplasmic DNases is implicated in cytoplasmic DNA accumulation and SASP in senescent cells. Nat Commun. 2018; 9:1249. https://doi.org/10.1038/s41467-018-03555-8 PMID:29593264

3. Tang Z, Li C, Kang B, Gao G, Li C, Zhang Z. GEPIA: a web server for cancer and normal gene expression profiling and interactive analyses. Nucleic Acids Res. 2017; 45:W98–102. https://doi.org/10.1093/nar/gkx247 PMID:28407145

4. Menyhárt O, Nagy Á, Győrffy B. Determining consistent prognostic biomarkers of overall survival and vascular invasion in hepatocellular carcinoma. R Soc Open Sci. 2018; 5. https://doi.org/10.1098/rsos.181006 PMID:30662724
