# Supplementary material for: DNASE1L3 arrests tumor angiogenesis by impairing the senescence-associated secretory phenotype in response to stress
Source: Aging (Albany NY). 2021 Mar 19;13(7):9874–99. doi: 10.18632/aging.202740 (PMC8064203; doi:10.18632/aging.202740)
Supplement: Supplementary Figures [file aging-13-202740-s002.pdf]

SUPPLEMENTARY FIGURES

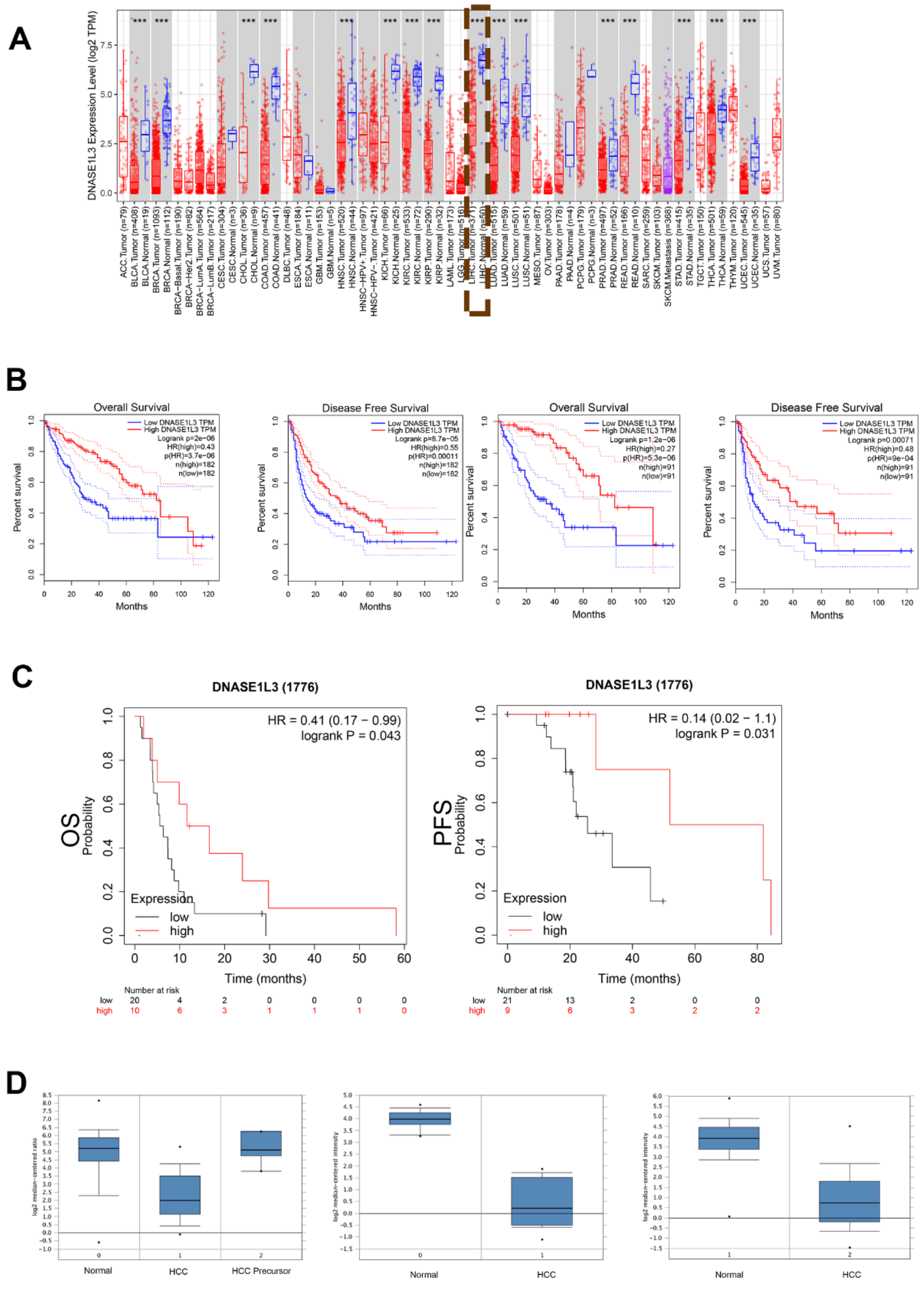

Supplementary Figure 1. (**A**) The mRNA of DNASE1L3 is low expressed in multiple types of tumors from TCGA database. The green dotted frame showed the expression of DNASE1L3 in Liver hepatocellular carcinoma (LIHC). (**B**) Kaplan-Meier curves for overall survival and disease-free survival in HCC patients from the TCGA database showed patients with higher mRNA expression of DNASE1L3 had a better prognosis than those with lower expression. (**C**) OS and PFS in cohort of patients treated with sorafenib, the results indicate low expression of DNASE1L3 is associated with lower insensitivity of sorafenib treatment. (**D**) The mRNA of DNASE1L3 is low expressed in multiple cohorts from Oncomine database. Left, Chen's cohort; Middle, Roessler's cohort1; Right, Roessler's cohort2.

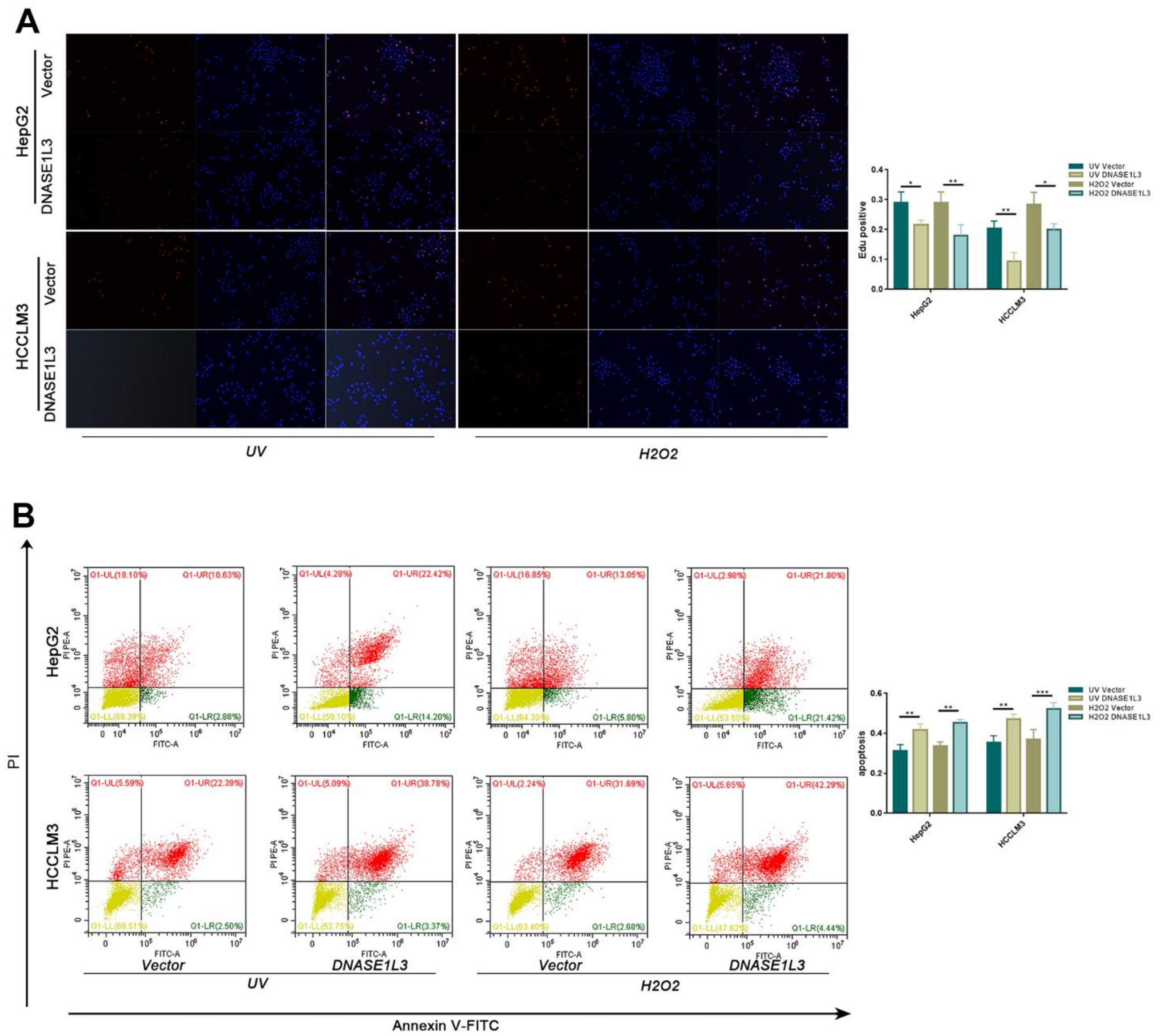

Supplementary Figure 2. (**A**) Reduced DNA synthesis in cells treated in different groups were assessed, representative images were shown (red, Edu; blue, DAPI; 10×). (**B**) Overexpression of DNASE1L3 promotes the apoptosis of cells post DNA damage in the early stage (data were obtained on day2 after DNA damage treatments).

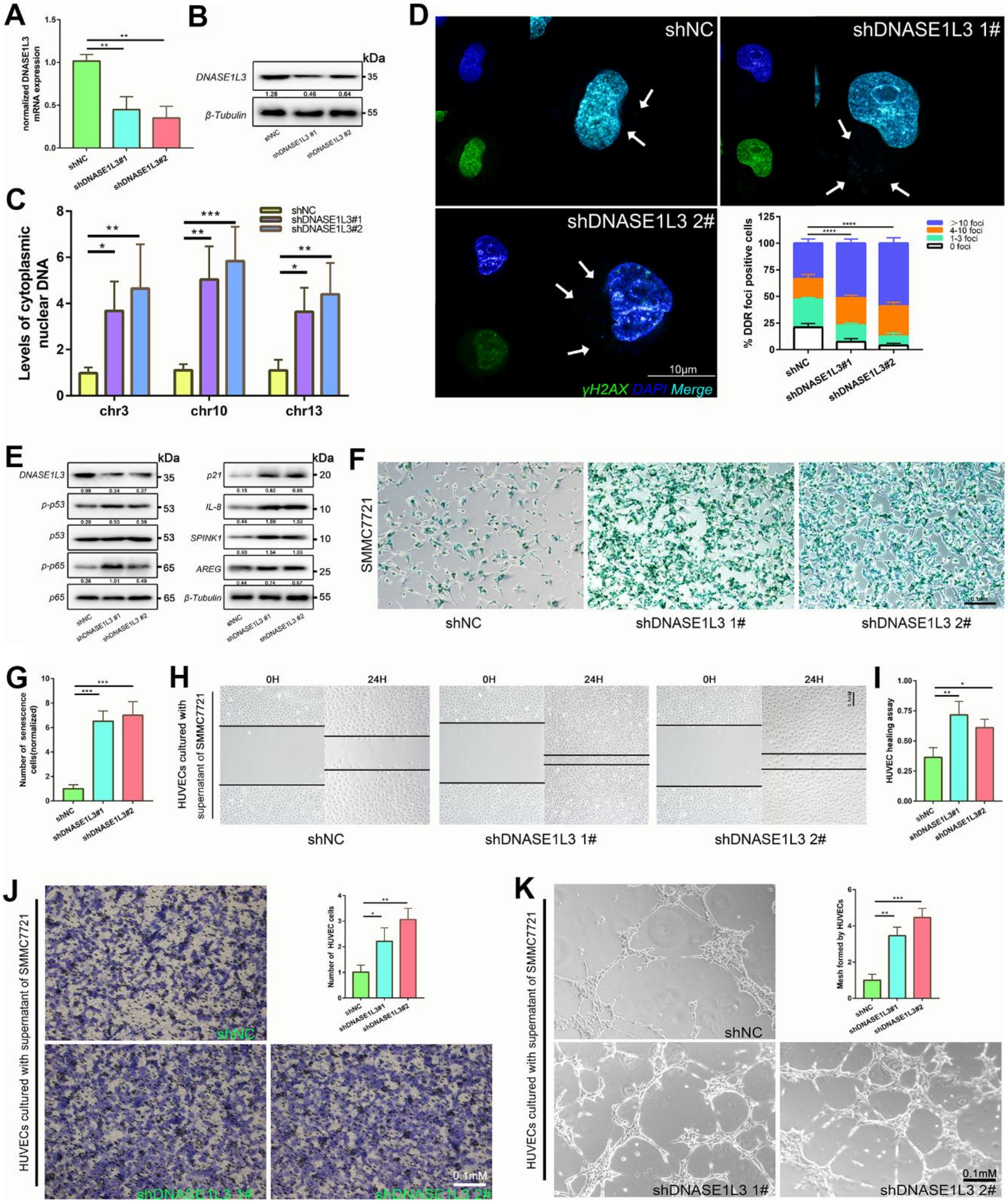

Supplementary Figure 3. (**A**, **B**) DNASE1L3 knockdown in SMMC7721 cell using shRNA(sh)-mediated interference. (**C**) qPCR analysis of chromosomal DNA in cytoplasmic fraction of cells treated in different group. (**D**) Cytoplasmic accumulation of nuclear DNA in differentially treated cells were assessed, representative images were shown (green, γH2AX; blue, DAPI; Scale bars, 10 μm). The number of DNA damage foci (DDF) per cell falls into each of the 0, 1-3, 4-10, and >10 counting categories when quantified the DDF. (**E**) Immunoblot analysis of inducible expression change of senescence associated signal pathway and downstream proteins including p53, p65, SPINK1 and AREG in different treated groups. (**F**) SA-β-Gal staining of cells in differently treated groups (scale bar, 200 μm). (**G**) Statistics of SA-β-Gal staining cells in differently treated groups. (**H**, **I**) The motility of HUVECs were assessed by wound healing assay, the supernatants from cells in differently treated groups were added into the culture of HUVECs, images were taken at 0h and 24h (scale bar, 100 μm). (**J**) The cellular migration ability of HUVECs were determined by the transwell migration assay. The supernatants from cells in differently treated groups were added into the lower chamber, images were taken after 24h of incubation (scale bar, 100 μm). (**K**) The tube formation ability of HUVECs were determined by tube formation assay. The supernatants from cells in differently treated groups were added into the culture, images were taken after 6h of incubation (scale bar, 100 μm). The results show the means ± SD from at least three separate experiments.

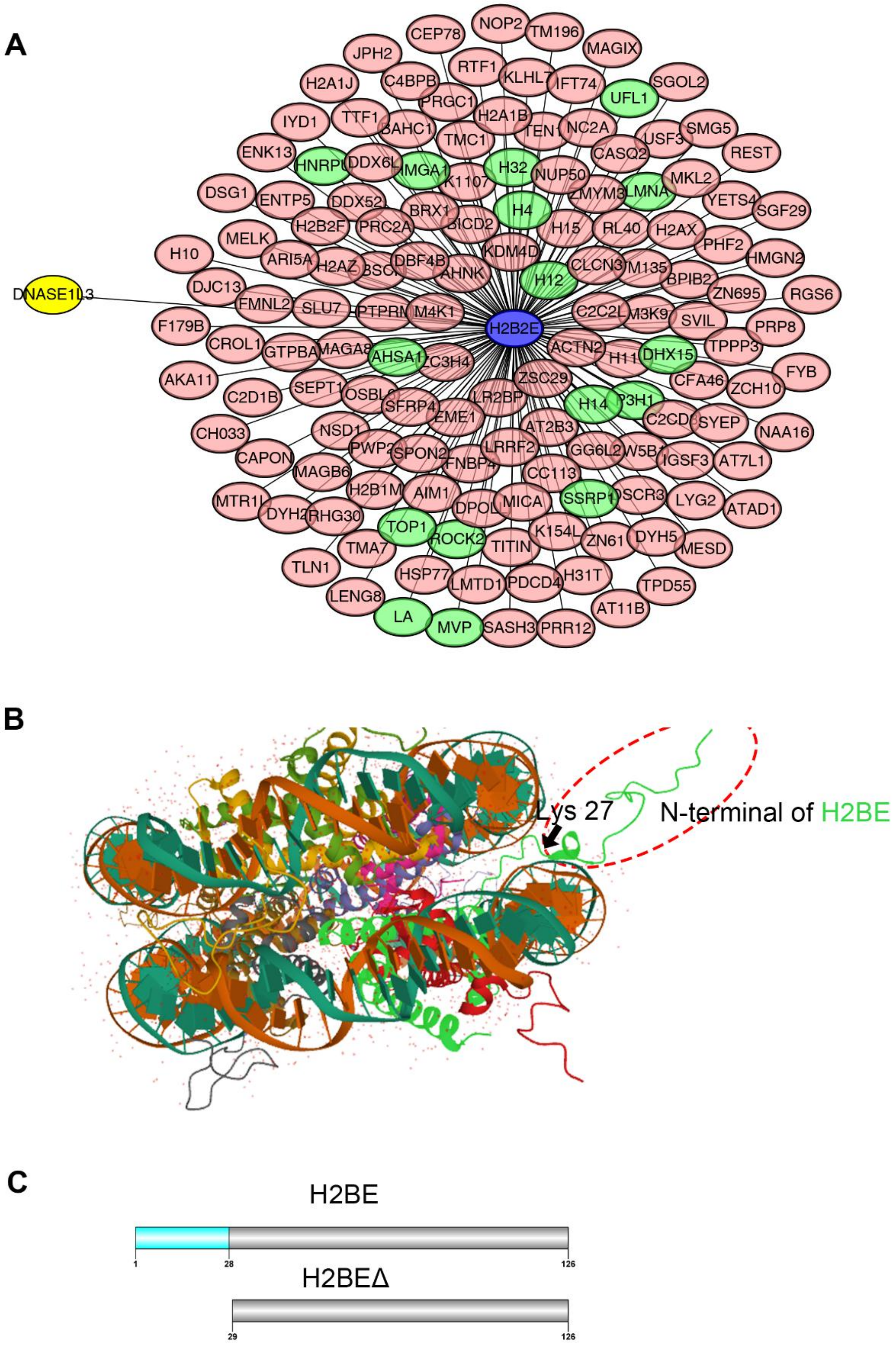

Supplementary Figure 4. (**A**) IntAct Molecular Interaction Database were analyzed (https://www.ebi.ac.uk/intact/interaction/EBI-20919708) and a PPI network was constructed. For the PPI network, network nodes represent proteins, and edges represent protein-protein associations. The DSSO crosslink assay show DNASE1L3 could binding to H2BE, and H2BE binding to other proteins. The nodes colored in green means the protein was also captured in our coIP-MS assay from another unpublished work. (**B**) The N-terminal region of H2BE (amino acids 1-28) is the area on the protein surface, and it is not winded by DNA sequence. The red dotted frame showed the N-terminal region of H2BE in the 3D model. The 3D model of H2BE is downloaded from RCSB database (https://www.rcsb.org/3d-view/1KX5). (**C**) Reconstructed H2BEΔ with the N-terminal region deleted.
