# Supplementary material for: DNASE1L3 arrests tumor angiogenesis by impairing the senescence-associated secretory phenotype in response to stress
Source: Aging (Albany NY). 2021 Mar 19;13(7):9874–99. doi: 10.18632/aging.202740 (PMC8064203; doi:10.18632/aging.202740)
Supplement: Supplementary Tables [file aging-13-202740-s003.pdf]

SUPPLEMENTARY TABLES

Supplementary Table 1. Tumor parameters of the DNASE1L3 high-expression and low-expression groups.

Parameters	DNASE1L3 expression		Total	*P* value
	High(n=102)	Low(n=102)		
**Age(y)**				0.376
≤55	64	70	134	
>55	38	32	70	
**Gender**				0.452
Male	83	87	170	
Female	19	15	34	
**HBV DNA load, (IU/ml)**				**0.007***
$\leq 10^4$	54	35	89	
$>10^4$	48	67	115	
**AFP (ng/mL)**				0.092
≤400	60	48	108	
>400	42	54	96	
**Liver cirrhosis**				**0.672**
yes	56	59	115	
no	46	43	89	
**Tumor size(cm)**				**<0.001***
≤5	65	23	88	
>5	37	79	116	
**No. of tumors**				**0.038***
Solitary	87	75	162	
Multiple	15	27	42	
**MVI**				**<0.001***
Presence	14	46	60	
Absence	88	56	144	
**Edmondson-Steiner classification**				**<0.001***
I-II	62	32	94	
III-IV	40	70	110	

* Significant results ($P < 0.05$) are given in bold.
Abbreviation: HBV, hepatitis B virus; MVI, microvascular invasion.

Supplementary Table 2. Univariate and multivariate analysis with a Cox proportional hazard regression model for overall survival.

variable	Univariate analysis			Multivariate analysis		
	HR	95%CI	*P* value	HR	95%CI	*P* value
**Age**	0.821	0.564-1.195	0.303	-	-	-
**Gender**	0.856	0.545-1.345	0.500	-	-	-
**HBV DNA load**	1.105	0.778-1.570	0.577	-	-	-
**AFP**	1.376	0.972-1.948	0.072	-	-	-
**Liver cirrhosis**	0.827	0.583-1.172	0.285	-	-	-
**Tumor size**	1.712	1.194-2.453	**0.003***	1.047	0.698-1.570	0.826
**No. of tumors**	2.122	1.406-3.202	**<0.001***	2.064	1.355-3.145	**0.001***
**MVI**	2.028	1.407-2.923	**<0.001***	1.636	1.101-2.430	**0.015***
**Edmondson-Steiner classification**	1.657	1.161-2.366	**0.005***	1.458	1.004-2.119	**0.048***
**DNASE1L3 expression**	0.378	0.264-0.540	**<0.001***	0.519	0.337-0.801	**0.003***

* Significant results (P < 0.05) are given in bold.
Abbreviations: HR, hazard risk ratio; CI, confidence interval.

Supplementary Table 3. List of the primers used in this study.

Target gene		Primer (5'-3')
GAPDH	F	GTCTCCTCTGACTTCAACAGCG
	R	ACCACCCTGTTGCTGTAGCCAA
DNASE1L3	F	TGGTTGAGGTCTACACGGACGT
	R	GTCAGTCCTCAAGCGGATGTTC
p16	F	CTCGTGCTGATGCTACTGAGGA
	R	GGTCGGCGCAGTTGGGCTCC
p21	F	AGGTGGACCTGGAGACTCTCAG
	R	TCCTCTTGGAGAAGATCAGCCG
IFN-β	F	CTTGGATTCCTACAAAGAAGCAGC
	R	TCCTCCTTCTGGAACTGCTGCA
CXCL10	F	GGTGAGAAGAGATGTCTGAATCC
	R	GTCCATCCTTGGAAGCACTGCA
IL-1β	F	CCACAGACCTTCCAGGAGAATG
	R	GTGCAGTTCAGTGATCGTACAGG
IL-6	F	AGACAGCCACTCACCTCTTCAG
	R	TTCTGCCAGTGCCTCTTTGCTG
IL-8	F	GAGAGTGATTGAGAGTGGACCAC
	R	CACAACCCTCTGCACCCAGTTT
Chr-3	F	TCAAGTGCCACATCCTATGC
	R	ATTTTTCTAGCCAGGCACCA
Chr-10	F	ACCTGGAAATGGCTGAAATG
	R	AAGTCCTCGCAGAGGTTTCA
Chr-13	F	CGCCAGTGTGTGTAGCACTT
	R	TCGGCCTCTCTCAGTTCTGT

Supplementary Table 4. List of the antibodies used in this study.

Antibody	Source	Dilutions
DNASE1L3	Abcam(ab203669)	IHC:1/100
DNASE1L3	Proteintech(67041-1-Ig)	WB: 1/500
Beta-Tublin	Proteintech(10094-1-AP)	WB: 1/1000
p-p53	Proteintech(28961-1-AP)	WB: 1/1000
p53	Proteintech(60283-2-Ig)	WB: 1/2000
p-p65	Abcam (ab76302)	WB: 1/1000
p65	Proteintech(66535-1-Ig)	WB: 1/1000
p21	Proteintech(60214-1-Ig)	WB: 1/500
IL-8	Abcam (ab110727)	WB: 1/1000
SPINK1	Abcam (ab207302)	WB: 1/5000
AREG	Abcam (ab213698)	WB: 1/1000
anti-Flag	Proteintech(20543-1-AP)	WB: 1/200
anti-C-Myc	Abcam (ab32072)	WB: 1/1000
H2BE	Abcam (ab1790)	WB: 1/1000
H3	Abclonal(A2348)	WB: 1/500
